# Outcomes of Surgical Excision and Brachytherapy in Intractable Keloids

**Published:** 2017-09

**Authors:** Ahmadreza Taheri, Hojjat Molaei, Mehdi Aghili, Naser Rahmanpanah, Atefeh Mirmohseni

**Affiliations:** 1 Plastic and Reconstructive Surgery Department, School of Medicine, Tehran University of Medical Sciences, Tehran, Iran; 2 Oncology and Radiotherapy Department, School of Medicine, Tehran University of Medical Sciences, Tehran Iran

**Keywords:** Brachytherapy, Excision, Surgery, Keloids

## Abstract

**BACKGROUND:**

Keloids as unusual scars are injury remnants characterized by bizarre cosmetics and painful itching. This study assessed outcomes of surgical excision and brachytherapy in intractable keloids.

**METHODS:**

Six patients with 10 keloid lesions were followed up. Surgical excision was done with 1-2 mm margin, and then radiotherapy was undertaken in 3 divided fractions on days 0, 1 and 2 after surgery. Scar improvement was evaluated by patients and observer with scar assessment scale (POSAS)

**RESULTS:**

Median age of patients was 38.3±6.4, while 40% were male and 60% were female. The mean primary size of the lesion before brachytherapy was 325.18±426.16 mm^2^ and the median size was 153.48 mm^2^. The mean primary size of the lesions with recurrence before brachytherapy was 150.50±124.78 mm^2^. The clinical improvement of the scars with POSAS scoring by the observer was 17.1±3.2 and by the patients was 20.8±11.5. In 5 patients who were evaluated, two keloid lesions showed recurrence (20%), and 8 lesions had no recurrence (80%). No patients reported side effects, but only one patient, a 43 years old woman with 5 keloid lesions, suffered wound infection and local dehiscence of the wound, followed by the second session of brachytherapy. The average time of relapse was 26.3±0.9 months.

**CONCLUSION:**

The use of surgical resection in combination with brachytherapy was demonstrated as a modality for treatment of refractory keloid scars that can be recommended to surgeons who deal with these patients.

## INTRODUCTION

Keloids as unusual scars are injury remnants characterized by bizarre cosmetics and painful itching.^1^ Disorganized collagen bundles plus uncontrollable production of fibroblast proteins result in poor quality scars as keloid.^2^ Keloids recurrent nature, even after surgery are challenges for the clients that welcomes adjuvant therapies. Radiation and brachytherapy- interstitial or internal radiation- can be assumed as one of more effective modalities to conquer the dilemma.^3^

High-dose-rate (HDR) brachytherapy is preferred over low-dose-rate (LDR) brachytherapy, because the patient can be treated as outpatient in contrast to hospitalization in LDR for 48-72 hours.^3^ Accordingly, we established a study to evaluate the efficacy of surgical excision plus HDR brachytherapy in patients treated in Imam Khomeini Hospital, a center for intractable keloids affiliated to Tehran University of Medical Sciences, Tehran, Iran.

## METHODS AND MATERIALS

Six patients with keloid lesions who were admitted for treatment from Jan 2009 to Dec 2013 entered our study. One patient was excluded due to deficient data and at last 5 patients with 10 keloid sites were followed up. Our inclusion criteria were (i) To be candidate for resection, (ii) To have pathological confirmation, (iii) To be unresponsive to current modalities for at least 1 year, (iv) Responded as the size decreased to 50% or regaining to 50 % primary size after surgical excision, and (v) Available complete census for surgical excision and HDR brachytherapy. 

Exclusion criteria in our study were (i) Pregnancy and lactation, (ii) Brachytherapy contraindication, (iii) Incomplete data and (iv) Not being cooperative. This retrospective study accomplished in Plastic and Reconstructive Ward of Valieasr Hospital and Cancer Institute of Imam Khomeini Hospital. Under general or local anesthesia, surgical excision was done with 1-2 mm margin, then a brachytherapy catheter (French-6) was inserted subdermally as 4-5 centimeter over both borders to have intact skin. Finally, the wound was closed with nylon 4-0 or 5-0 sutures and the catheter fixed the skin using silk 4-0.

Patients were transferred to radiotherapy ward, 30 to 90 minutes after operation and the catheter was connected to the machine to receive radiation in less than 30 min. Total dose was 12 Gy that was delivered in 3 divided fractions on days 0, 1 and 2 after surgery and then the catheter was removed. 

Scar improvement was evaluated by the patients and the observer based on scar assessment scale (POSAS),^4^ the items including scar color, pliability, thickness, healing, itching, pain and patient`s general feeling and observer items including scar vasculature, pigmentation, thickness , healing, pliability, scar area and general condition were determined. Scoring was 1 to 10 which 1 denoted to normal and 10 to the worst condition. The Patients were evaluated on days 7, 14 and then every 3 months on first year, and later every 6 months, while the questionnaires were completed on these time intervals. The collected data were analyzed using SPSS software (Version 20, Chicago, IL, USA).

## RESULTS

**Fig. 1:**
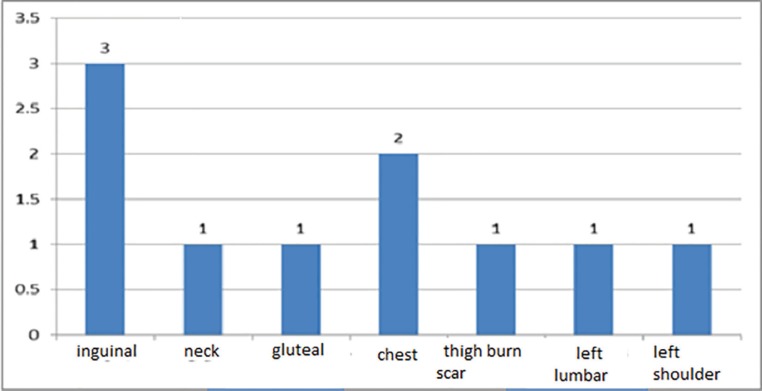
The frequency of keloid lesions areas

**Fig. 2:**
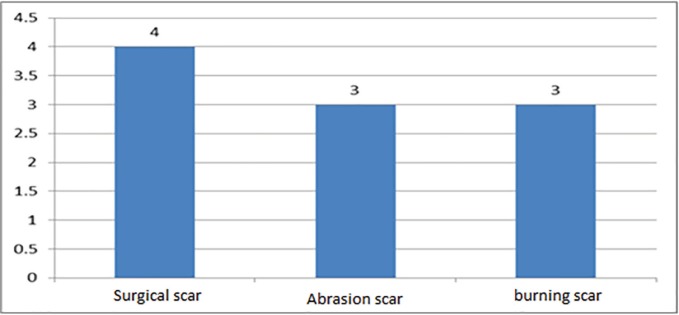
The etiology of the keloid lesions

Among the 5 patients with 10 keloid lesions reviewed, the median age was 38.3±6.4 years (From 32 to 49 years old). Two patients were male (40%) and the remained 3 were female (60%). The frequency of keloid lesion areas was 3 in the inguinal region, 1 in the neck, 1 in the gluteal area, 2 in the chest and the right clavicle (shoulder), 1 burn scar in thigh, 1 in the left lumbar and 1 was in the left shoulder (Figure 1). The etiologies of the keloid lesions in 4 cases (40%) were surgical scars, in 3 (30%) were abrasion scars and in 3 cases (30%) were burn scars (Figure 2).

**Fig. 3:**
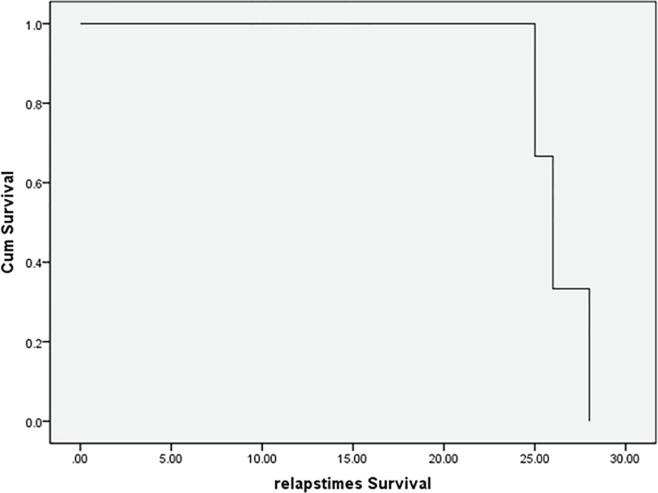
The average time of relapse

**Fig. 4:**
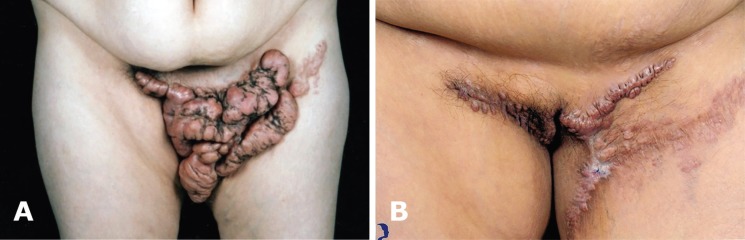
**A-B:** Before and after surgical resection and brachytherapy (Patient 1).

The mean primary size of the lesion before brachytherapy was about 325.18±426.16 mm^2 ^and the median size was 153.48 mm^2^. The mean primary size of the lesions with recurrence before brachytherapy was 150.50±124.78 mm^2^. The clinical improvement of the scars based on POSAS scoring by the observer was 17.1±3.2 and by the patients was 20.8±11.5. In 5 patients, two keloid lesions revealed recurrence (20%), and 8 lesions did not have any recurrence (80%). No patients reported any side effects, except one patient, a 43 year old woman with 5 keloid lesions, suffered from wound infection and local wound dehiscence, followed by the second session of brachytherapy. The average time of relapse was 26.3±0.9 days (Figures 3 and 4).

## DISCUSSION

The main concern of doctors and patients following surgery is unpredictable scar formation and so any slight improvement of the scars seems to be marvelous. In recent years, various therapeutic methods, medical and surgical methods have been used to improve keloid scars, but these interventions have not yield optimal results.^5^ High recurrence rate is the most highlighted concern, and there is not any accepted as gold standard method for keloid scar treatment. One of the relatively successful methods in refractory keloid treatments is combination of surgical resection and brachytherapy.^6^

Brachytherapy alone or LDR brachytherapy +surgery have been undertaken showing the rate of recurrence in 1 year follow up to be between13 and 27%. However, in HDR brachytherapy, the recurrence rate has been less than 5%.^3^ According to our data, the keloid lesions ratio of relapse was 20% during 2 years follow up and was not associated with any recurrence after the year 2^nd^. Patient satisfaction on aesthetic results and clinical symptom relief, such as pruritus, pain, and other complaints, was above 80%, whereas according to other studies for simple surgical resection, the recurrence has been more than 80%.^7^^,^^8^

In a research of intralesional corticosteroid injection treatment, the response rate has been 50% over a period of 5 years.^9^ Other therapeutic options included physical interventions such as pressure garment after surgery, laser therapy, silicone gel, intralesional INF injection and also intralesional 5-FU injection. None of them revealed any role (real impact) in preventing scar formation, while the recurrence rate after treatment was beyond 50%.^10^ Meanwhile, it has been shown that brachytherapy in combination with surgery, can be assumed as an effective treatment with various recurrence rates from 3.5 to 27% depending on the level of radiation.^11^^,^^12^

Due to different brachytherapy protocols in our study, and also the location of the keloid scars, the recurrence rates have been diverse. Nevertheless, it seems that combination of surgical treatment+brachytherapy can control the rate of recurrence, better than any other managements and it can be considered as the treatment choice for keloid lesions, which is available, nonexpensive and convenient for the patient. High dose brachytherapy was more effective in preventing recurrence of keloid scars and besides, HDR brachytherapy alone was an applicable method for patients who declined surgery.

In a study, 35 Patients with 54 keloids were treated postoperatively with HDR brachytherapy (1*4 Gy+2*3 Gy), with only one recurrence out of 35 denoting to the effectiveness of HDR brachytherapy after keloidectomy.^13^ In another research, early results of high-dose-rate brachytherapy after keloidectomy were evaluated in 21 patients with 36 keloids, 3 keloids in 2 patients had local recurrence (9.7%) with a median time of 12 month, while our study had decreased rates with 20% of relapse rate and median time of failure was after 26 month.^14^^,^^15^

Our patients had no major complications following brachytherapy and none of them complained for pain or any discomfort from catheterization. Therefore, catheter was well tolerated during the 3-4 days of brachytherapy and it could be easily removed without anesthesia at the end of treatment. The important advantages of the prescribed protocol in our research were the acceptable cosmetic results, low recurrence rate, low complication rates and its long-term treatment effect. Since our routine brachytherapy method was dealing LDR with IR192, 12 Gy in divided doses of 3 Gy in every session, resulted into a recurrence ratio of 20%, so it is possible to decrease the recurrence rate as much as 5% like other similar studies, by increasing the radiation dosage in the range of 18-20 Gy. 

According to the findings in this research and similar studies, surgical resection in combination with high dose brachytherapy in treating keloid scars, was associated with significant advantages including to be painless, an easy and inexpensive procedure, to be done in outpatient settings without no major contraindication, a high local control, excellent patient tolerance, proper distribution of radiation dose and lastly, to have the radiation as the lowest level upon normal tissues.

Therefore, applying surgical resection in combination with brachytherapy as a treatment modality for treatment of refractory keloid scars, is our recommendation to all professionals and surgeons who deal with these patients. However, further studies are necessary to support this hypothesis, with greater number of patients and keloid lesions and long-term follow up to evaluate the long-term recurrence rate, Moreover, the possibility of radioactive mutagens` side effects in the remaining scars needs to be assessed.
